# Under-representation of male patients in breast cancer studies

**DOI:** 10.1186/1745-6215-16-S2-P110

**Published:** 2015-11-16

**Authors:** Pankaj Mistry, Louise Hiller, Gulnaz Iqbal, Janet Dunn

**Affiliations:** 1 University of Warwick, Coventry, West Midlands, UK

## 

Latest Cancer Research UK figures state that approximately 350 men are diagnosed with breast cancer each year compared to 50,000 women. There are many benefits for patients who participate in Randomised Controlled Trials (RCT), including access to new drugs or interventions before they are available on the NHS. However, males appear to be underrepresented in breast cancer RCTs. Research was carried out to assess the potential reasons and implications of this.

A brief literature review was undertaken of recently published phase III RCTs of breast cancer to assess the eligibility criteria with respect to gender and, if both sexes were eligible, the ratio of males to females in the final trial population.

Of ten recently published papers, five categorically state only women were eligible, four infer that only women were eligible but do not explicitly state it, , and one omits mention of sex in the eligibility section however all trial patients were women. Two of the four papers that inferred only women were eligible mention the use of mammogram equipment which is not designed to screen male patients.

Various reasons exist behind the upfront exclusion of men from RCTs, for example, assessments may include mammograms or trial endpoints may focus heavily on cosmesis or gender related QoL measures. However, the efficacy of treatments and toxicities suffered may vary between gender groups. Therefore, both genders need to be represented in order to ensure generalisability to the wider UK population. Further investigation is required to determine the size of the problem.

